# Berberine-Drug Interactions: Mechanisms, Clinical Relevance and Risk Stratification—A Narrative Review

**DOI:** 10.3390/ph19081313

**Published:** 2026-08-20

**Authors:** Caterina Nela Dumitru, Teodora Marcu, Alina Oana Dumitru, Simona Steliana Tudor, Ionela Daniela Ferțu, Alina-Mihaela Elisei, Larisa Goroftei

**Affiliations:** 1 Research Centre in the Medical-Pharmaceutical Field, Faculty of Medicine and Pharmacy, “Dunărea de Jos” University of Galați, 35 Al. I. Cuza Street, 800010 Galați, Romania; 2 Faculty of Medicine and Pharmacy, “Dunărea de Jos” University of Galați, 35 Al. I. Cuza Street, 800010 Galați, Romania

**Keywords:** berberine, supplement–drug interactions, cytochrome P450, P-glycoprotein, QT prolongation, pharmacomicrobiomics, pharmacovigilance, clinical pharmacy, food-supplement regulation

## Abstract

**Background:** Berberine, an isoquinoline alkaloid present in *Berberis* spp., *Coptis chinensis* and *Hydrastis canadensis*, is among the most widely consumed metabolic-health supplements, popularized as “nature’s Ozempic”. Concurrent, often undisclosed use with prescription drugs is common in older adults, yet berberine is far from inert. **Objective:** To synthesize the evidence on berberine as a perpetrator of supplement–drug interactions, propose a four-axis mechanistic taxonomy, with product quality treated separately as a modifier of exposure rather than as a mechanism, and derive a clinically actionable risk-stratification framework. **Methods:** Structured narrative review, prepared per the SANRA quality criteria; PubMed/MEDLINE, Scopus, Web of Science and Embase were searched up to May 2026. **Results:** Despite very low systemic exposure (oral bioavailability 0.68% in rats; low ng/mL plasma concentrations in humans), high luminal, enterocytic and hepatic concentrations generate interaction liability, documented in humans for a few pairs and mechanistic for most, along four mechanistic axes: inhibition, and transcriptional induction, of CYP3A4, with CYP2D6/CYP2C9 inhibition that is quasi-irreversible through a metabolite-intermediate complex; transporter modulation (P-glycoprotein, OCT1/OCT2, and MATE1); pharmacodynamic additivity (hypoglycemia, hypotension, and QT prolongation); and microbiome- and gut-barrier-mediated effects, the last of these being a candidate axis rather than a demonstrated one. Product-quality variability is treated separately, as a modifier of exposure. The clinical anchor is increased cyclosporine exposure in renal-transplant recipients (AUC +34.5%; trough 29.3% above control). These elements are integrated into a three-tier risk-stratification framework that combines perpetrator potency, victim-drug vulnerability, and patient vulnerability, with each tier being linked to a defined pharmacy action. **Conclusions:** In patients on multiple medications, and particularly when berberine is co-administered with drugs of narrow therapeutic index, it should be managed as an active pharmacological perpetrator rather than as an inert supplement. Unstandardized product quality and an unsettled European regulatory framework, under which national limits differ by more than an order of magnitude, further widen the uncertainty around the dose actually delivered. Berberine use should therefore be elicited routinely at medication reconciliation and stratified by mechanism, by victim-drug vulnerability, and by patient risk, with particular attention to metabolic self-medication in the GLP-1 era.

## 1. Introduction

Interactions between foods or dietary supplements and drugs are an underappreciated source of therapeutic variability and iatrogenic risk [1,2]. Unlike drug–drug interactions, those involving “natural” products are frequently omitted at history taking, because of the perception that what is natural is harmless [1,3]. Population studies report that between 5% and over 80% of older adults combine supplements with prescription drugs, and a substantial proportion carry at least one potential interaction [3,4,5,6,7]. The failure of patient–clinician communication about supplement use amplifies this risk, as physicians rarely initiate the discussion [1,3]. The historical benchmark of food–drug interactions remains grapefruit juice, whose furanocoumarins irreversibly inhibit intestinal CYP3A4 and increase exposure to numerous drugs [8,9,10]. This model demonstrated that a common dietary component can have clinically relevant pharmacokinetic consequences [10,11].

Over the past decade, the rise in supplements for metabolic health has shifted this problem from an academic concern to a public-health one [12,13]. Berberine, a quaternary isoquinoline alkaloid isolated from *Berberis vulgaris*, *Coptis chinensis*, *Hydrastis canadensis* (goldenseal) and *Phellodendron* spp., occupies a particular position in this landscape [14,15,16,17]. On the one hand, it is supported by a consistent body of evidence for hypoglycemic and lipid-lowering effects, mediated through multiple mechanisms: activation of AMP-activated protein kinase (AMPK) [18,19,20,21], increased insulin-receptor expression [22], reduction in PCSK9 [23] and regulation of non-coding RNAs [24]. Meta-analyses of randomized trials confirm significant reductions in glycated hemoglobin, fasting glucose and lipid profile with berberine [25,26,27,28], and its metabolic efficacy has been compared, in some syntheses and combination clinical studies, with that of common oral hypoglycemic agents [29,30,31]. On the other hand, its popularization as “nature’s Ozempic” on social media has generated a wave of unsupervised use [12,13].

This enthusiasm coexists with two structural vulnerabilities [32,33]. The first is product quality: botanical identity and actual alkaloid content vary considerably, and contamination and substitution are documented in botanical products [32,34]. The second is berberine’s pharmacology itself, which is far from inert [35,36]. Berberine inhibits major cytochrome P450 enzymes and modulates membrane transporters essential to drug disposition [35,36,37]; it also exerts pharmacodynamic effects that can add to those of numerous therapeutic classes [38,39,40]. Nonetheless, the clinical literature dedicated to berberine interactions remains fragmented, dispersed across isolated pharmacokinetic studies, case reports, and broad pharmacological reviews in which interactions are treated only marginally [2,41].

It should be stated at the outset that berberine interactions are not a novel topic: recent syntheses have specifically addressed cytochrome P450 inhibition by berberine and barberry [42], and comprehensive reviews have already tabulated the main reported drug–drug interactions [2,43,44]. The present contribution does not claim priority in describing these mechanisms; rather, it reorganizes them into a unified taxonomy and, more importantly, extends the framework beyond enzymes and transporters. We thus add four elements absent from earlier syntheses: (i) a taxonomy of mechanisms, across four axes, with product identity and quality treated separately as a modifier of exposure rather than as a mechanism, that turns scattered observations into a clinical reasoning framework; (ii) a pharmacomicrobiomic axis proposed as a candidate interaction mechanism rather than only an efficacy mechanism, with its present evidential limits stated; (iii) an operational risk-stratification framework intended for the clinical pharmacist; and (iv) positioning of the problem within the multiple metabolic self-medication specific to the era of glucagon-like peptide-1 (GLP-1) receptor agonists, which we treat as a research priority rather than as a documented interaction [45,46,47].

## 2. Results and Discussion

### 2.1. Pharmacokinetic Features of Berberine Relevant to Interactions

Understanding berberine’s interaction potential begins with an apparent paradox [43]. The absolute oral bioavailability of berberine is very low, estimated to be below 1% in rats and below a few percent in humans [48,49,50], a consequence of its physicochemical properties and of the limited absorption consistently documented in the literature [51,52,53] of P-glycoprotein-mediated efflux and of extensive intestinal and hepatic first-pass metabolism [48,54,55]. The peak plasma concentration after usual doses is on the order of nanograms per milliliter, frequently below the activity threshold observed in vitro [49,56], and tissue distribution favors the liver and intestine over plasma [57]. Such low systemic exposure would seem to imply a minimal interaction risk; the opposite holds, and for a reason intrinsic to the poor absorption itself [43]: berberine reaches high intraluminal and enterocytic concentrations, exactly at the level where absorption and first-pass metabolism of orally administered drugs occur [48,54]. Local inhibition of CYP3A4 and P-glycoprotein in the intestinal wall can markedly increase the absorbed fraction of the victim drug, independently of berberine’s modest plasma concentrations [36,58]. Chronic, repeated administration, typical of supplement use, lets these inhibitory effects accumulate, and it carries a time-dependent component of CYP2D6 inhibition that we characterize in Section 2.2.1 [35,59]. Berberine and its metabolites (berberrubine, thalifendine, demethyleneberberine, and jatrorrhizine), formed predominantly by CYP1A2, CYP2D6 and CYP3A4, are themselves substrates and/or inhibitors of several enzymes and transporters, which broadens the spectrum of interactions [59,60,61].

The size of that gap between systemic and local exposure decides which in vitro potencies are relevant at all. A usual oral dose of 400–500 mg gives peak plasma concentrations of a few nanograms per milliliter, which for a molecule of about 336 Da is roughly 1–10 nM [49,56]. The same dose dispersed in the 200–300 mL of fluid available in the upper small intestine gives a nominal luminal concentration close to 5 mM, four to six orders of magnitude higher. Enterocyte and portal concentrations lie between these extremes, and liver and intestinal tissue levels stay above plasma throughout the absorption phase [48,54,57]. Both figures should then be read against the potencies collected in Table 1, which cluster between 1 and 45 µM: luminal concentrations exceed that band by two to three orders of magnitude, while plasma concentrations fall below it by two to four. Inhibition that needs micromolar exposure is therefore realistic at the intestinal wall and unrealistic in the systemic circulation, which is why the same compound behaves as a strong perpetrator for orally absorbed substrates and a weak one where disposition is purely systemic. The luminal value is an order-of-magnitude estimate and not a measurement: poor aqueous solubility, pH-dependent speciation, transit time, food and precipitation all move it, so the local concentration has to be modeled rather than assumed.

Quantifying the local exposure that truly matters for interactions cannot be inferred from plasma concentrations but requires dedicated methods [48,62]. Standardized in vitro simulated gastrointestinal digestion protocols reproduce the salivary, gastric and intestinal stages and allow for estimation of the fraction of bioactive compounds that remain stable and bioaccessible along the tract [62]. Applied to berberine, they help separate the fraction acting luminally, relevant to inhibition of enzymes and transporters at the intestinal wall, from the systemically absorbed fraction [48,54]. Because transporter-level interactions depend on local concentration, integrating bioaccessibility data with physiologically based pharmacokinetic (PBPK) models represents the most promising methodological direction for quantitative prediction of berberine interactions [63,64].

**Figure 1:**
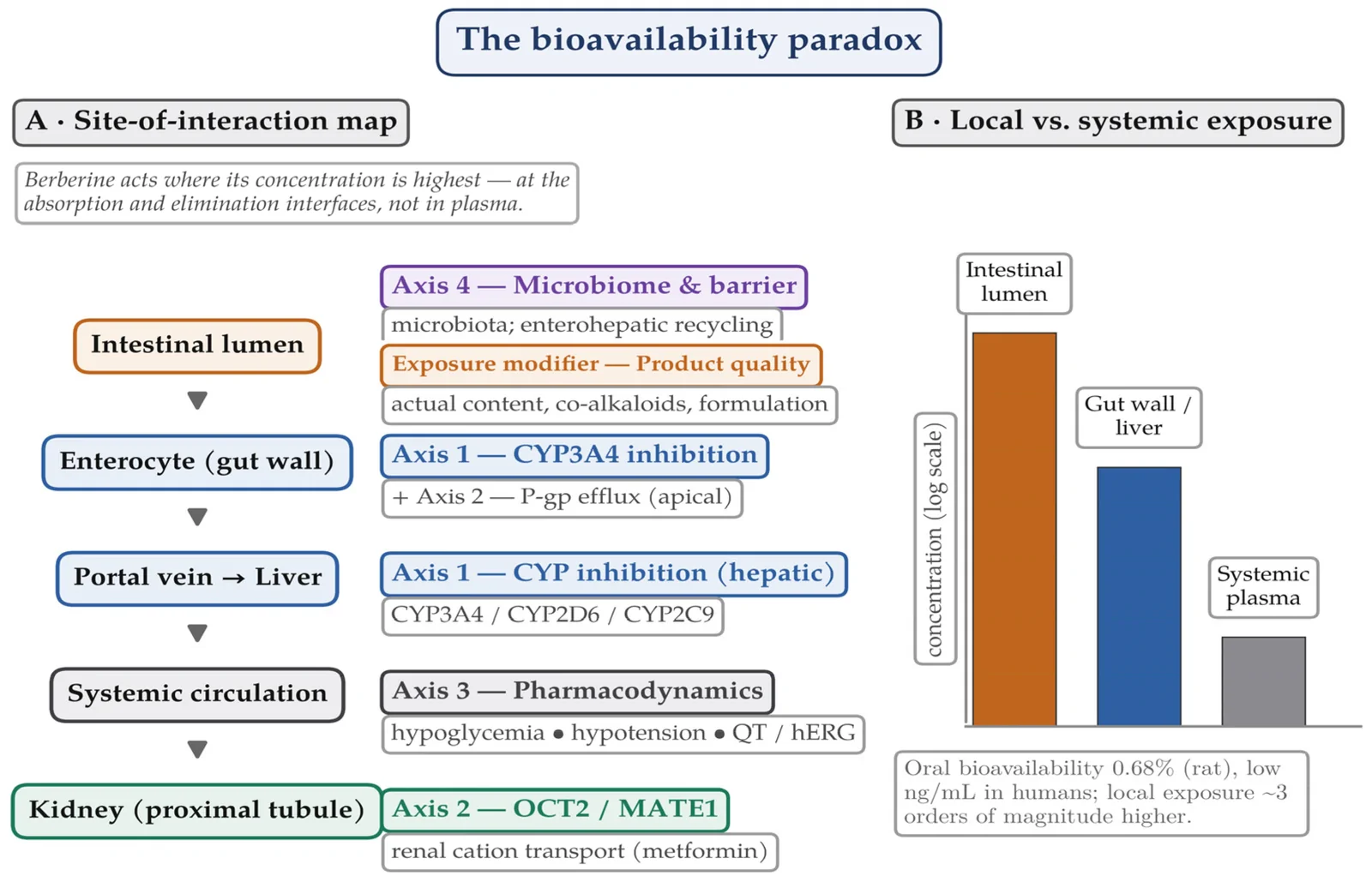
Site-of-interaction map and the bioavailability paradox. Despite low systemic bioavailability, berberine reaches concentrations orders of magnitude higher at the sites of absorption (intestinal lumen, enterocyte) and elimination (liver, kidney), where the majority of interactions occur [37,43,48]. Only the boxes numbered Axis 1 to Axis 4 are mechanistic axes of the taxonomy. The box labeled Exposure modifier—Product quality is deliberately left unnumbered: product identity, declared versus actual alkaloid content, and formulation are not a biological mechanism of interaction but a cross-cutting modifier that sets the perpetrator dose on which all four mechanistic axes operate (Section 2.2.5). It is drawn at the intestinal-lumen level because that is where the delivered dose first becomes relevant, and its position immediately below Axis 4 carries no hierarchical meaning. Panel (**A**) maps the successive compartments through which orally administered berberine passes—intestinal lumen, enterocyte, portal vein and liver, systemic circulation, and renal proximal tubule—and aligns each compartment with the mechanistic axis that operates at that site; the grey downward triangles indicate the direction of this transit and carry no quantitative meaning. Panel (**B**) compares, on a logarithmic scale, the concentrations attained in the intestinal lumen, at the gut wall and liver, and in systemic plasma; it is schematic and not drawn to scale. Original figure by the authors.

At the renal level, berberine is transported vectorially through organic cation transporter 2 (OCT2) at the basolateral membrane and through multidrug and toxin extrusion protein 1 (MATE1) at the apical membrane, while also being a substrate of P-glycoprotein [37]. This dependence on organic cation transporters explains why berberine competes with cationic drugs eliminated by the same route [37,65]. In addition, an important fraction of the dose is metabolized by the intestinal microbiota into better-absorbed forms, such as dihydroberberine, which links pharmacokinetics to the state of the microbiome [43,66]. In summary, low systemic exposure does not protect against interactions; rather, it shifts their site toward the absorption and elimination interfaces, where local concentrations are high (Figure 1) [37,43,48].

### 2.2. Taxonomy of Berberine’s Interaction Mechanisms

We propose organizing berberine interactions along four complementary mechanistic axes, to which we add, separately, one modifier of exposure that is not itself a mechanism, in order to turn a scattered list of observations into a clinical reasoning framework [41,67]. Each victim drug can thus be classified according to the dominant mechanism and the anatomical site of the interaction [2,64].

Throughout this section, and in Table 1 and Table 2, the strength of the evidence is reported with the grading scheme set out in full in Section 3: Grade A, a controlled clinical study using the victim drug itself; Grade B, a clinical study in humans with probe substrates, enzyme-activity or pharmacodynamic endpoints; Grade C, an in vivo preclinical study; Grade D, in vitro or purely mechanistic data, physiologically based pharmacokinetic simulation included; and Grade E, no direct data, the effect being inferred from mechanism. Case reports are graded separately, since they establish that an event occurred without quantifying its frequency or its magnitude. The four axes are not equally well supported, and it is better to say so before the reader invests in the framework. Axis 1 rests on a human enzyme-phenotyping study and on a clinical probe study with a goldenseal extract, so its inhibition limb reaches Grade B, while its induction limb is Grade D. Axis 2 has one Grade A anchor, cyclosporine in renal-transplant recipients, one Grade B human probe study for metformin, and Grade C or D evidence for everything else. Axis 3 has Grade B pharmacodynamic evidence for glucose and blood pressure, but its cardiac component is Grade D supplemented by case reports. Axis 4 has nothing above Grade D for any drug pair and is presented as a candidate. Read this way, the taxonomy is a map of where the evidence sits, not a claim that the four axes carry equal weight.

#### 2.2.1. Axis 1—Inhibition and Induction of Cytochrome P450 Enzymes

The best-documented axis is enzyme inhibition [35,36]. In a clinical study in volunteers, repeated administration of berberine reduced CYP2D6, CYP2C9 and CYP3A4 activity, a human enzyme-activity finding, Grade B, that provides the mechanistic basis for the potential interactions discussed below [35]. In vitro, berberine inhibits CYP2D6 with greater potency (reported IC50 values on the order of 7–45 µM) and CYP3A4/CYP2C9 with lower potency [36,68,69]. CYP2D6 inhibition is quasi-irreversible in the strict pharmacological sense rather than simply long-lasting, and the primary source describes it as such: the effect develops in a time-, concentration-, and NADPH-dependent manner and is attributed to formation of a metabolite-intermediate complex (MIC) with the heme iron [59]. Berberine carries a methylenedioxyphenyl group, the structural motif that classically supports this chemistry, and the criteria that separate an MIC from true irreversible inactivation are spectral, an absorbance maximum near 455 nm, and functional, partial recovery of activity on treatment with potassium ferricyanide. The distinction matters for what follows. The complex is tight enough that activity does not return once the unchanged alkaloid has been cleared, so the inhibition outlives systemic exposure to berberine; it is not, however, a covalent adduct, and recovery in vivo should track enzyme resynthesis rather than dissociation. What nobody has measured is how long CYP2D6 activity stays suppressed in humans after berberine is stopped, which means the clinical duration of this component is inferred from the in vitro mechanism and not observed [35,59]. This CYP2D6 relevance is reinforced by the fact that this isoenzyme, together with CYP1A2, is also the main metabolizing pathway of berberine itself; CYP2D6 polymorphism and sex-related differences influence berberine exposure, adding a source of interindividual variability in the magnitude of interactions [70]. Because these enzymes collectively metabolize a major proportion of commonly used drugs, the spectrum of potential victims is broad: CYP3A4 substrates (statins, calcium-channel blockers, immunosuppressants, and some benzodiazepines), CYP2D6 substrates (many antidepressants, antipsychotics, beta-blockers, codeine, and tramadol) and CYP2C9 substrates (warfarin, some anti-inflammatories, and sulfonylureas) [2,35,36].

Clinical relevance is illustrated by goldenseal-based products, a source of berberine and hydrastine [71,72]. Goldenseal supplementation markedly inhibited the metabolism of midazolam, a CYP3A probe substrate, with an increase in exposure on the order of 40–60% [72,73]. The mechanism involves formation of a metabolic-intermediate complex with CYP3A, described for the methylenedioxyphenyl components of goldenseal [74]. In fact, (−)-β-hydrastine appears to be the main alkaloid responsible for this time-dependent inhibition, berberine being a weaker CYP3A4 inhibitor; therefore, the alkaloid profile and botanical identity of the product influence the magnitude of the interaction [71,74]. Physiologically based pharmacokinetic models also predict increased exposure to CYP3A substrates such as imatinib in the presence of goldenseal alkaloids [63].

An aspect often overlooked is that berberine’s profile is not exclusively inhibitory. Recent data show that berberine and its metabolite berberrubine can induce, in a concentration-dependent manner, the expression of CYP3A4 through activation of the nuclear pregnane X receptor (PXR) [75]. The coexistence of inhibition, acute and, for CYP2D6, quasi-irreversible, with possible transcriptional induction upon chronic administration explains why the net direction of the effect on a CYP3A4 victim may depend on dose, duration of exposure, and tissue; this phenomenon calls for caution in the linear extrapolation of in vitro inhibition data to the clinical situation [75].

The practical question is when this induction begins and at what concentration. Berberine and berberrubine activate PXR at micromolar concentrations in the in vitro systems used, the same range as the enzyme-inhibition IC50 values reported above and three to four orders of magnitude above the plasma concentrations set out in Section 2.1 [75]. Onset follows the kinetics of PXR-mediated transcription rather than anything peculiar to berberine. For prototypical PXR ligands, CYP3A4 mRNA rises within roughly 6 to 24 h of exposure; protein and catalytic activity follow over about 2 to 3 days, limited by turnover of the existing enzyme pool; a new steady state is reached after some 1 to 2 weeks of repeated dosing, and de-induction takes a comparable amount of time after withdrawal. Two consequences follow for berberine. Inhibition should dominate the first days of use, while induction can only express itself after a week or more of uninterrupted supplementation, which is exactly the pattern of supplement use and exactly the window no study has examined. And because the effective concentrations are micromolar, induction is plausible in the enterocyte and in the hepatocyte exposed to portal blood, but not in the systemic circulation, so an effect on intestinal CYP3A4 is more likely than one on hepatic CYP3A4. We give these figures as extrapolations from PXR biology in general: neither the onset time nor a concentration–response relationship has been measured for berberine in humans, and the point is carried forward in Section 2.5 [63,75].

#### 2.2.2. Axis 2—Modulation of Membrane Transporters

The second axis concerns efflux and uptake transporters, recognized components of drug disposition [64,76]. Berberine is both a substrate and an inhibitor of P-glycoprotein (P-gp/MDR1), and can increase the intestinal absorption of its substrates [58,77,78], a principle also exploited in TPGS-type formulations that increase berberine’s own intestinal absorption [41]. In preclinical models, berberine increases the bioavailability of digoxin and cyclosporine, two classic P-gp substrates, through inhibition of intestinal P-gp; for digoxin the increase in exposure was dose-dependent and occurred only after intragastric, not intravenous, administration, consistent with an intestinal site of interaction [79,80]. Berberine’s effect on P-gp expression is, however, tissue- and cell-line-dependent, with induction reported in certain contexts [77,79]. Because P-gp and CYP3A4 are frequently colocalized in the enterocyte and share substrates, simultaneous inhibition of both can have a multiplicative effect on bioavailability [58,64].

This colocalization is particularly relevant for immunosuppressants with a narrow therapeutic index. A 2025 pharmacokinetic study in rats showed that different administration regimens of berberine hydrochloride alter exposure to sirolimus, a CYP3A/P-gp substrate, supporting extension of the interaction signal from cyclosporine to the whole class of calcineurin and mTOR inhibitors [81].

At the level of organic cation transporters, berberine inhibits OCT1, OCT2 and MATE1 in a concentration-dependent manner, with half-maximal inhibitory concentrations of 18.8, 1.02 and 10.7 µM, respectively [65]. The three are not interchangeable, and the discussion that follows stays with OCT2 and MATE1 because that is where the in vivo evidence sits. OCT1 is expressed mainly on the sinusoidal membrane of the hepatocyte, with lower expression in the enterocyte, and it governs entry of cationic drugs into the liver rather than their elimination. For metformin the distinction is not academic, since the liver is the site of action and not a clearance organ: inhibition of OCT1 would be expected to reduce hepatic uptake and blunt the glucose-lowering effect while leaving systemic concentrations unchanged or even higher, a dissociation between exposure and effect that plasma monitoring would miss. Berberine is also the weakest inhibitor of the three at OCT1, with an IC50 roughly eighteen times that at OCT2, so at the concentrations reached in portal blood, this is the least likely of the three mechanisms to be expressed. It has not been tested directly, and we keep it here as a mechanistic possibility rather than a documented interaction [65,82]. This mechanism underlies a bidirectional interaction with metformin, a prototypical substrate of these transporters. In the terms set out in Section 3 this is the one transporter-mediated pair that passes beyond mechanistic status: it is documented in animals and, for systemic exposure, in a human probe study [65,82,83]. In in vivo models, berberine reduced the peak plasma concentration and exposure of metformin and increased its renal accumulation, through inhibition of OCT/MATE-mediated transport [65]. The direction of the effect is route-dependent: after intravenous administration of metformin, berberine increased exposure and reduced clearance, indicating that the net result reflects the balance between inhibited absorption and inhibited renal secretion [82]. Clinical support comes from a probe-cocktail study in 16 healthy volunteers, in which a goldenseal extract standardized to berberine reduced metformin systemic exposure while increasing midazolam AUC (geometric mean ratio 1.43; 90% CI 1.35–1.53) [83]. The interaction illustrates an important principle: the net effect on a victim depends on the location of the affected transporter (absorption versus elimination) and cannot be predicted from in vitro inhibitory potency alone [64,65]. Berberine also reduced the oral bioavailability of ciprofloxacin in animal models, suggesting involvement of organic anion/cation transporters [78]. A semi-quantitative synthesis of these effects is presented in Table 1.

**Table 1:** Semi-quantitative synthesis of berberine’s inhibition and induction of enzymes and transporters. Numerical values are given only where explicitly reported in the primary sources. AUC = area under the concentration–time curve; APD = action-potential duration; NR = not reported; IC50 = half-maximal inhibitory concentration; and PXR = pregnane X receptor. Letters in parentheses in the Experimental System column are the evidence grades defined in Section 3 and applied identically in Table 2: B = clinical study in humans with probe substrates, enzyme-activity or pharmacodynamic endpoints; C = in vivo preclinical study; and D = in vitro or purely mechanistic data. Case reports are labeled separately, since they establish occurrence but not magnitude. ↑ = increase.

Target	Type of Effect	Reported Potency/Magnitude	Experimental System	Reference
CYP2D6	Inhibition, quasi-irreversible (metabolite-intermediate complex)	IC50 ≈ 7–45 µM	Human liver microsomes; volunteers (D and B)	[35,36,59,69,70]
CYP3A4	Inhibition and induction (PXR)	↑ midazolam AUC ≈ 40–60% (goldenseal); induction in vitro	Clinical (phenotyping); in vitro (B and D)	[36,72,73,74,75]
CYP2C9	Inhibition	Lower potency; IC50 NR quantitatively	Human liver microsomes (D)	[35,36]
CYP1A2	Substrate/inhibition	NR quantitatively	In vitro (D)	[61,69,70]
P-gp (MDR1)	Substrate + inhibitor	↑ bioavailability of digoxin, cyclosporine, sirolimus (preclinical)	Caco-2; in vivo models (D and C)	[55,58,77,79,80,81]
OCT1/OCT2	Inhibition	IC50 18.8 (OCT1)/1.02 (OCT2) µM	Transfected cell lines; in vivo (D and C)	[65,82,83]
MATE1	Inhibition	IC50 10.7 µM	Renal vectorial transport (rat) (C)	[37,65]
hERG (IKr)	Channel block	IC50 ≈ 3.1 µM (HEK-293); ≈75–80 µM (oocytes); APD/QT prolongation	Electrophysiology; case reports (D plus case reports)	[40,84,85,86]

#### 2.2.3. Axis 3-Pharmacodynamic Additivity

Beyond pharmacokinetics, berberine exerts pharmacodynamic effects that can dangerously add to concomitant medication [40]. Its intrinsic hypoglycemic effect adds to that of antidiabetic agents, such as sulfonylureas and insulin, increasing the risk of hypoglycemia [30,38]. Vasodilatory and bradycardic effects can potentiate the hypotension and bradycardia induced by antihypertensives and beta-blockers [40,87], hemodynamic effects documented even in patients with severe heart failure [88].

The most concerning signal is the cardiac electrophysiological one [39,89]. Berberine blocks the hERG channel that carries IKr, with only weak effects on the KCNQ1/KCNE1 channels underlying IKs, and prolongs the action-potential duration and the QT interval [84,86]. A recent systematic review of berberine-associated arrhythmias confirms that, although it also has antiarrhythmic properties, it can produce QT prolongation, bradycardia and hypotension [89]. Cases of torsades de pointes associated with berberine use have been reported. In a published case report, acquired long QT syndrome with torsades de pointes (QTc 667 ms) occurred in a woman taking 250 mg berberine together with a cannabidiol/cannabigerol hemp-oil preparation at six times the recommended dose; the QTc normalized within five days of stopping all supplements. Causality cannot be assigned to berberine alone, since cannabidiol both prolongs repolarization and inhibits CYP3A4 and CYP2D6, which makes this case an illustration of, rather than an exception to, the combined mechanism described below [90]. A further signal has been reported at the conference-abstract level; the small number of cases warrants caution in interpretation [39]. The risk increases when berberine is combined with other drugs that prolong QT or that inhibit its metabolism, producing a pharmacokinetic and pharmacodynamic “double hit” [39,91]; experimentally, concomitant administration of berberine and macrolides enhances cardiac toxicity, providing a direct preclinical model of this combination [92]. A caveat must nevertheless be stated explicitly: reported potencies for direct hERG block differ markedly between expression systems, with an IC50 of approximately 3.1 µM in mammalian HEK-293 cells but approximately 75–80 µM in *Xenopus* oocytes [84,86]; even the lower of these values exceeds, by two to three orders of magnitude, the plasma concentrations achieved after usual oral doses, so acute IKr block cannot on its own explain a clinical QT signal. Two mechanisms may reconcile the discrepancy: trafficking-dependent reduction in hERG surface expression, which develops over prolonged exposure and at far lower concentrations [85], and pharmacokinetic amplification when berberine is combined with inhibitors of its own metabolism or with other QT-prolonging agents. Both remain unquantified in humans, and the cardiac axis should accordingly be read as a plausible, mechanistically anchored but clinically uncalibrated risk. This places berberine within the broader group of drugs and supplements with a risk of induced arrhythmia, monitorable through pharmacovigilance, in which the pharmacist’s assessment has a preventive role [91,93,94].

#### 2.2.4. Axis 4-Microbiome- and Gut-Barrier-Mediated Effects (Pharmacomicrobiomics): A Candidate Axis

The fourth axis, frequently omitted in discussions of berberine interactions, derives precisely from its therapeutic mechanism [43,95]. Because most of the dose remains in the intestinal lumen, berberine remodels the composition of the microbiota and has broad-spectrum antimicrobial activity [95,96]. Berberine enriches short-chain fatty-acid-producing bacteria, modulates bile-acid metabolism and remodels the microbiota in models of obesity and insulin resistance [95,97,98,99,100,101]; the microbiome in turn biotransforms it into better-absorbed forms [43,66,102].

The relevance to interactions arises from the fact that the microbiome is a general mediator of xenobiotic metabolism, capable of activating, inactivating or recycling numerous drugs [103,104,105]. Therefore, berberine can indirectly influence the disposition of other drugs by at least three routes: altering bacterial drug metabolism, modifying the enterohepatic circuit through regulation of bacterial β-glucuronidases, and modulating gut-barrier integrity [95,104,105]. Through its intrinsic antimicrobial activity and its high luminal concentrations, berberine is a prototypical example of a non-antibiotic compound that perturbs the intestinal microbial ecosystem [43,106]. It is worth being explicit about what this evidence does and does not establish. The three routes above are documented for the intestinal microbiota in general; none has been demonstrated for berberine acting on a named co-administered drug. The available work reports what berberine does to community composition, not what that change does to the disposition of anything else. We therefore present this axis as a candidate mechanism rather than a demonstrated one, and it should not carry the weight of Axes 1 to 3 in clinical reasoning. What would settle the question is a study measuring the pharmacokinetics of a microbiota-dependent victim drug before and after berberine, with the microbiota characterized in the same subjects; we set this out in Section 2.5 [104,105,106].

**Figure 2:**
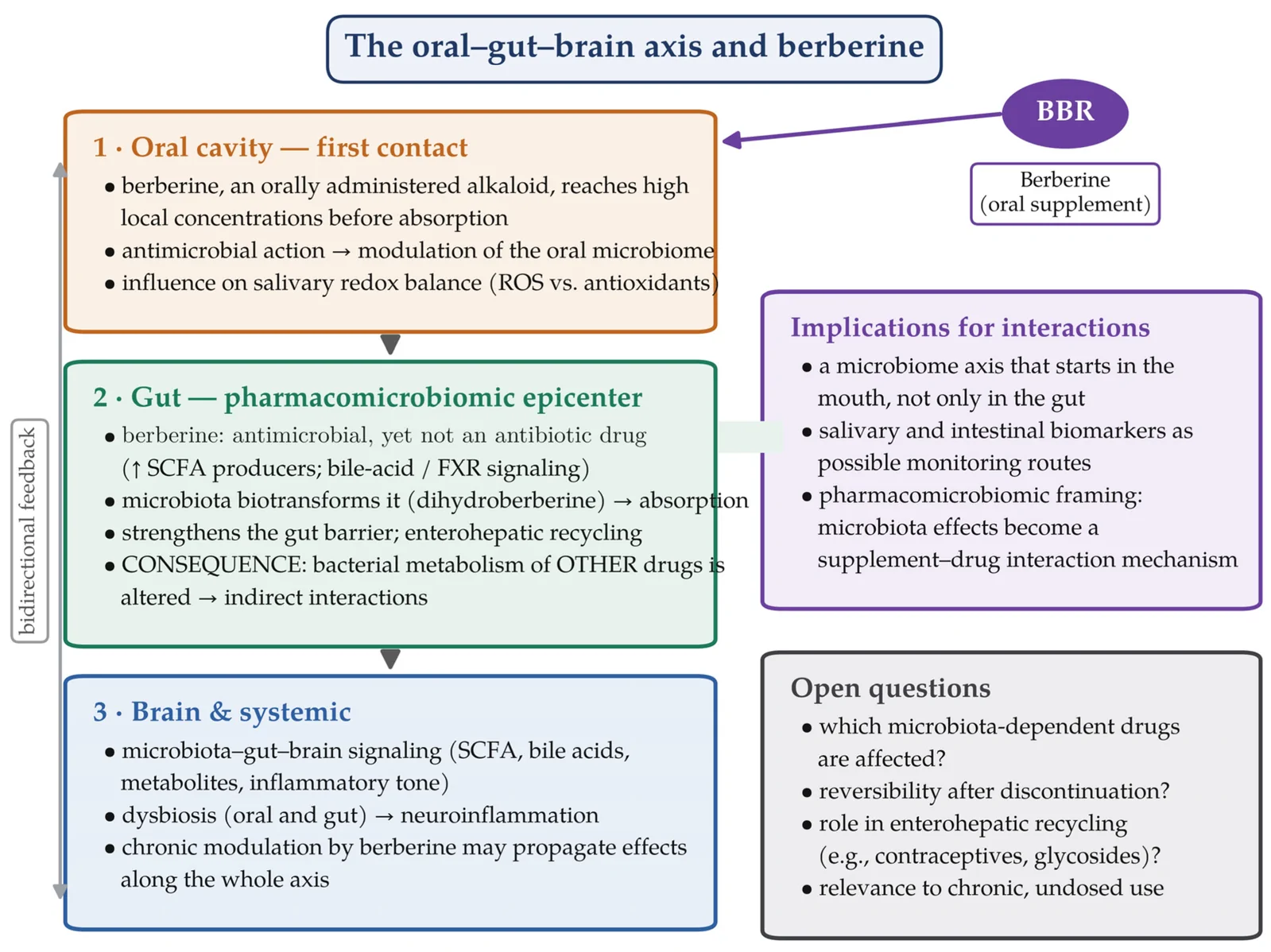
Extension, at the hypothesis level, of the microbiome axis from the oral cavity to the oral–gut–brain axis, and the pharmacomicrobiomic implications of berberine [106,108]. The limb relevant to drug interactions is the oral-to-intestinal one; the neural limb is shown as physiological context only. The purple arrow marks the entry of orally administered berberine at the oral cavity. The grey downward triangles indicate the direction of progression along the oral–gut–brain axis, and the grey double-headed arrow on the left denotes bidirectional feedback between compartments. Within the boxes, ↑ denotes an increase and → denotes a consequence. The colours of the boxes distinguish the three compartments (orange, oral cavity; green, gut; blue, brain and systemic) and carry no quantitative meaning. Original figure by the authors.

One extension of this axis needs its interaction rationale stated plainly, because it is not self-evident. The mouth is the first microbial compartment that a swallowed supplement meets, and oral bacteria carry reductive and hydrolytic activities of the same type that make the intestinal microbiota a determinant of drug disposition [103,104,105]. Berberine reaches high local concentrations there before absorption and, being broadly antibacterial, can act on that community [107]. Whether this alters the fate of anything co-administered has not been tested, so we leave the oral compartment as a research question and state it in Section 2.5 rather than developing it here. Figure 2 should be read from the mouth to the gut; the neural arm is physiological context and not an interaction mechanism [108].

#### 2.2.5. A Cross-Cutting Modifier of Exposure: Variability in Product Identity, Quality and Regulatory Status (Not a Mechanistic Axis)

This last component sits deliberately outside the mechanistic taxonomy. It is not a biological mechanism and does not belong among the axes; it is the modifier that determines the dose on which all four mechanisms operate, and we present it separately for that reason [32]. The actual berberine content, the presence of co-alkaloids such as hydrastine, the chemical form, and “enhanced-bioavailability” formulations determine the effective perpetrator dose the patient receives [71,109,110]. Botanical contamination and substitution are documented through DNA barcoding techniques in botanical products [34]. The toxicity of berberine and related alkaloids also depends on dose and formulation [33,111], and the gastrointestinal safety profile is documented in dedicated syntheses [112]. Non-conformity of a significant proportion of marketed products means that the magnitude of any interaction is intrinsically unpredictable without standardization [32,34]. For the clinician, this modifier imposes a pragmatic rule: in the absence of a standardized, independently tested product, exposure should be treated as uncertain in both directions rather than simply higher-than-declared. Quantitative analysis of commercial berberine preparations found actual content deviating widely from the labeled amount [32], and surveys of botanical supplements describe the same two-sided pattern, with products that contain less-than-declared, as well as products that contain more, alongside substitution and undeclared co-alkaloids [2,34]. The two directions fail differently. A product containing more than the label states inflates any interaction beyond published estimates; one containing less produces apparent therapeutic failure and tempts the prescriber to dismiss the supplement altogether, even though the quantity present may still be enough to inhibit enzymes and transporters at the intestinal wall, where the relevant concentrations are local rather than systemic. Neither direction can be read off the label, so the defensible position is that the perpetrator dose is unknown [2,32,34].

One determinant of that dose lies outside pharmacology altogether. Berberine is sold across Europe as a food supplement, but it is not authorized on uniform terms: food-supplement law is harmonized only for vitamins and minerals, under Directive 2002/46/EC [113], while botanicals remain a national competence exercised through mutual recognition and through the procedure for other substances laid down in Article 8 of Regulation (EC) No 1925/2006 [114]. National limits consequently differ by more than an order of magnitude, from a cap of 10 mg per day on isoquinoline alkaloids in Belgium to no maximum dose in France and outright non-authorization in other Member States [115], so that the same nominal supplement can deliver a fiftyfold different daily dose depending on where it was bought. The position is also moving in one direction: ANSES identified pharmacological effects from 400 mg/day, could derive no toxicity reference value and set only an indicative value of about 0.1 mg/day for a 60 kg adult, and named drug interactions explicitly as a reason for clinician vigilance [115]; a subsequent EFSA draft opinion, still pending when this review was completed, concluded that no safe intake level could be established for the berberine-containing plant preparations examined [116]. Two points follow for the pharmacological argument made here. An unsettled regulatory status is itself a source of exposure variability, compounding the product-quality problem rather than replacing it; the interaction liability cataloged in this review is already among the stated grounds on which a national agency has asked prescribers to be vigilant [115], which puts medication reconciliation for berberine on a firmer footing than clinical habit alone.

### 2.3. Victim Drugs and the Strength of the Evidence for Each Pair

Table 2 summarizes the main victim-drug classes, the dominant mechanism, the likely direction of effect, and the graded level of evidence [35,37,39]. The grades are worth reading before the effect sizes, because only one row reaches the top of the scale. Emphasis is placed on drugs with a narrow therapeutic index, where even moderate changes in exposure can have clinical consequences [64,117]. The anchor evidence is the interaction with cyclosporine, demonstrated clinically in renal-transplant recipients. In a pharmacokinetic substudy (*n* = 6), 12 days of berberine 0.2 g three times daily increased cyclosporine AUC by 34.5% and reduced apparent oral clearance by 40.4%. In the parallel controlled trial (*n* = 104), trough concentrations rose by 88.9% from baseline in the berberine group; because the berberine-free group also rose by 64.5% from baseline, the between-group difference at study end was 29.3% for trough concentration and 27.8% for the concentration/dose ratio [118]. A separate study in healthy volunteers confirmed the direction of the interaction [117].

**Table 2:** Selected victim drugs, mechanisms and level of evidence. AUC = area under the concentration–time curve; Cmax = peak plasma concentration; Cmin = trough concentration; GI = gastrointestinal; and PK = pharmacokinetic. Levels of evidence, defined in Section 3 and applied identically in Table 1: A = controlled clinical study using the victim drug itself; B = clinical study in humans with probe substrates, enzyme-activity or pharmacodynamic endpoints; C = in vivo preclinical study; D = in vitro or purely mechanistic data; and E = no direct data, inferred from mechanism. Case reports are labeled separately, since they establish occurrence but not magnitude. ↑ = increase; ↓ = decrease.

Drug/Class	Dominant Mechanism	Likely Effect	Level of Evidence
Cyclosporine	CYP3A4 + P-gp inhibition [36,80]	↑ exposure (AUC +34.5%; Cmin +29.3% vs. control) [118]	A—controlled clinical study in patients (renal transplant) [117,118]
Tacrolimus, sirolimus	CYP3A4 + P-gp inhibition [58,80]	↑ exposure (sirolimus preclinical; tacrolimus extrapolated)	C—in vivo preclinical (sirolimus); tacrolimus by extrapolation, PBPK-supported [63,81]
Midazolam (CYP3A probe)	CYP3A4 inhibition (goldenseal) [72,73]	↑ AUC ≈ 40–60% [73,74]	B—clinical probe-substrate study (goldenseal) [72,73]
Metformin	OCT1/OCT2/MATE1 inhibition [65,82]	Bidirectional PK effect (↓ Cmax/AUC, ↑ renal accumulation); hypoglycemia risk via pharmacodynamic additivity [30,65]	B and C—clinical probe-cocktail study plus in vivo preclinical [65,82,83]
Sulfonylureas, insulin	Pharmacodynamic additivity [38]	↑ hypoglycemia risk [30]	B—clinical, pharmacodynamic endpoint [30,38]
GLP-1 receptor agonists	Additivity (glycemia, gastric emptying) [47]	↑ hypoglycemia/GI risk	E—no direct data; inferred from mechanism [45,46]
Warfarin	CYP2C9/CYP3A4 inhibition [36]	↑ anticoagulant effect possible	D—in vitro/mechanistic only; no victim-drug study [35,36]
Statins (simvastatin, atorvastatin)	CYP3A4 ± P-gp inhibition [36]	↑ exposure; ↑ myopathy risk	D—in vitro/mechanistic only; no victim-drug study [35,36]
Digoxin	P-gp inhibition [79,80]	↑ bioavailability	C—in vivo preclinical [80]
CYP2D6 substrates (beta-blockers, antidepressants, codeine, tramadol)	Quasi-irreversible CYP2D6 inhibition [59]	Altered exposure/effect	B—clinical, enzyme-activity endpoint; no victim-drug outcome [35,59]
QT-prolonging drugs	hERG block + metabolic inhibition [40]	↑ long-QT/torsades de pointes risk	D plus case reports—in vitro mechanism and published cases [40,84,90]
Antihypertensives/beta-blockers	Hemodynamic additivity [87]	↑ hypotension/bradycardia	B—clinical, pharmacodynamic endpoint [87,88]

### 2.4. A Proposed Risk-Stratification Framework for Pharmacy Practice

The central practical contribution of this review is a risk-stratification framework that integrates three multiplicative dimensions [2,64]. The first is berberine’s potency as a perpetrator on the relevant mechanism, derived from enzyme- and transport-inhibition data [35,36,65]. The second is the vulnerability of the victim drug, particularly a narrow therapeutic index [64,117]. The third is patient vulnerability, which includes age, renal and hepatic function, cardiac comorbidities, and pregnancy [93,111]. Table 3 operationalizes these dimensions into three risk levels, with corresponding actions [2,93].

Four operational principles complete the framework [1,2,93]. First, berberine must be actively recorded in medication reconciliation, through explicit questions about metabolic supplements [1,3]. Second, in the absence of a standardized product, exposure is assumed to be unknown in both directions, since the amount actually present may be lower or higher than the label states [32,33]. Third, temporal separation of administration reduces, but does not eliminate, absorption-level interactions and has no effect on systemic enzyme inhibition [54,59]. Finally, any suspected adverse event should be reported to the pharmacovigilance system, since reporting of supplement–drug interactions remains deficient [1,2]. Because controlled clinical interaction studies remain rare, detecting safety signals for berberine–drug pairs depends, in practice, on spontaneous reporting and disproportionality analyses in pharmacovigilance databases, which underscores the importance of structured recording of supplement use [91,119].

The three dimensions of this framework do not rest on the same kind of evidence, and the framework is more useful if that is visible. Perpetrator potency is the best-supported: enzyme inhibition has been measured in humans, Grade B, while the transporter component is largely in vitro, Grade D. Victim vulnerability rests on the established pharmacology of the victim drugs rather than on anything berberine-specific, which is a strength, since narrow therapeutic indices are not in dispute. Patient vulnerability is the weakest of the three: age, renal and hepatic function, and cardiac comorbidity are plausible modifiers, but no study has stratified a berberine interaction by any of them, so this dimension is expert interpretation and should be read as such. Within Table 3, the High risk row is anchored on Grade A evidence for cyclosporine alone; the other drugs in that row are placed there by mechanism and by the consequences of being wrong, not by direct data. We prefer to state this openly rather than let the table imply a uniform evidential basis it does not have.

### 2.5. Knowledge Gaps and Research Agenda

- High-risk drug pairs. Controlled clinical pharmacokinetic studies in humans are lacking for the berberine–victim-drug pairs at the highest theoretical risk, such as tacrolimus, warfarin and QT-prolonging substrates [39,63].
- Time course of enzyme modulation. The contribution of quasi-irreversible CYP2D6 inhibition, the balance between CYP3A4 inhibition and PXR-mediated induction, the concentration–response relationship for that induction and the time at which it begins, and the duration of both effects after berberine is stopped, all need to be quantified in humans; at present each is extrapolated from in vitro systems [59,75].
- Gut microbiome and drug metabolism. No study has yet measured the disposition of a co-administered drug before and after berberine while characterizing the microbiota in the same subjects, which is what would move Axis 4 from candidate to demonstrated. The oral compartment, where berberine reaches its highest concentrations of all, is unexamined and belongs in the same program [103,104,105,106,107,108].
- Product quality and reporting. Product standardization and reporting of actual alkaloid content in clinical studies are essential for reproducibility [32,34].
- Metabolic self-medication in the GLP-1 receptor agonist era. Patients on semaglutide or tirzepatide who add berberine on their own initiative are a population nobody has studied, and the combination has three theoretical dimensions, none of them tested. The first is pharmacodynamic: berberine’s hypoglycemic effect adds to that of the agonist and of any background antidiabetic agent [30,38]. The second is pharmacokinetic: GLP-1 agonists slow gastric emptying and so alter the absorption rate of oral drugs [47,120], while berberine inhibits enzymes and transporters at the same intestinal interface, so the two perturb one process from different directions [46,47]. The third is nutritional: reduced caloric intake under GLP-1 therapy can coexist with micronutrient deficiencies [121,122] that berberine might accentuate through transporter modulation [65,121]. Because supplement use is systematically under-reported [1,3], the first step is a prevalence estimate, followed by a targeted pharmacokinetic study; until then this belongs on the research agenda and not among the documented interactions, although it is already reason enough to ask any patient on GLP-1 therapy about metabolic supplements [45,46,47,123].
- Local-concentration PBPK models. Physiologically based pharmacokinetic (PBPK) models should be developed and validated to predict berberine interactions starting from local, not merely plasma, concentrations [63,64].
- Translational validation. Dedicated translational research is needed to connect mechanistic interaction signals with measurable clinical outcomes [119].

### 2.6. Limitations of the Review

As a narrative review, the present work does not include an aggregated quantitative assessment and is more exposed to selection subjectivity than a registered systematic review [124,125]. An important part of the mechanistic evidence comes from in vitro and preclinical studies, in which berberine concentrations may exceed human systemic exposure [48,68]; the clinical transferability of these data must therefore be interpreted with caution [64,68]. Finally, the heterogeneity of commercial products limits the generalizability of any effect-size estimate [32,33]. These limitations are inherent to the field and underscore the need for the proposed research agenda [125].

Several limitations deserve a fuller statement than the paragraph above gives them. Prospective clinical interaction studies are almost absent. Across the whole field we identified one controlled study in patients, in renal-transplant recipients receiving cyclosporine, one probe-cocktail study in healthy volunteers, and one human enzyme-phenotyping study; everything else is preclinical, in vitro or observational. A synthesis resting on three human studies cannot support strong statements about effect size in practice, whatever the mechanistic literature suggests [35,83,118].

Publication bias works in a particular direction here. Studies that find an interaction are more likely to be written up than those that do not, and negative pharmacokinetic results for supplement pairs rarely reach print at all, so the published record probably overstates both the frequency and the size of berberine’s effects. Case reports carry a different asymmetry. The torsades de pointes case discussed in Section 2.2.3 involved concurrent cannabidiol at six times the recommended dose, which is typical of supplement case reports, where polypharmacy and product uncertainty usually prevent attribution to a single agent [90]. Under-reporting to pharmacovigilance systems pushes the other way, and we cannot say which of the two dominates [91,119].

Product heterogeneity limits transferability more than the earlier paragraph implies. Formulations designed to raise absorption, whether TPGS-based, dihydroberberine or other enhanced-bioavailability preparations, change systemic exposure deliberately, so an effect size measured with one product cannot be carried over to another even when the labeled dose matches. Anyone applying the numbers in Table 1 and Table 2 should check which preparation generated them [32,41,109,110].

The translational gap, finally, is not incidental to this field but constitutive of it. The in vitro concentrations that produce inhibition exceed human plasma concentrations by two to four orders of magnitude, as Section 2.1 sets out; the local concentrations that would bridge the gap have been calculated but not measured in humans; the PBPK models that would connect the two have not been validated against clinical data for berberine; and the human endpoints available are surrogates, enzyme activity, or probe exposure, rather than clinical outcomes. Every step of that chain is plausible and none of it is closed, which is why this review argues for recording and monitoring rather than for quantitative dose adjustment [48,63,64,68].

## 3. Materials and Methods

This work is designed as a structured narrative review, with transparent reporting of the documentation strategy, guided by the quality criteria of the Scale for the Assessment of Narrative Review Articles (SANRA) [124]. The narrative approach is justified by the methodological heterogeneity of the evidence, which brings together in vitro mechanistic studies, preclinical models, clinical pharmacokinetic and pharmacodynamic studies, case reports, and regulatory documents that are difficult to aggregate quantitatively [125]. Consequently, no meta-analysis was performed; the aim was integrative synthesis, construction of a taxonomy of mechanisms, and identification of knowledge gaps [125].

Two conventions used throughout deserve stating explicitly, because the vocabulary of this field is loose enough to inflate certainty on its own. The first is a grading scheme for the strength of evidence, applied in Table 1 and Table 2 and referred to in the text. Grade A is a controlled clinical study using the victim drug itself. Grade B is a clinical study in humans with probe substrates, enzyme-activity or pharmacodynamic endpoints. Grade C is an in vivo preclinical study. Grade D is in vitro or purely mechanistic data, and we count physiologically based pharmacokinetic simulation here as well, since a PBPK prediction is a model built on in vitro inputs and not an independent observation. Grade E is no direct data at all, the effect being inferred from mechanism. Case reports are graded separately: they establish that an event occurred without quantifying how often or how large it is.

The second convention is terminological. A mechanistic interaction is one demonstrated only in vitro or in silico. A potential interaction is one where the mechanism is established and co-exposure is plausible, but no human study of that specific pair exists. A documented interaction has been measured in humans, by either a pharmacokinetic or a pharmacodynamic endpoint. A clinically significant interaction is a documented one whose magnitude warrants a change in monitoring or dose. On that reading, one pair in this review is clinically significant, a small number are documented, and most are potential or mechanistic. The unqualified word interaction refers to the general phenomenon and carries no claim about evidence.

PubMed/MEDLINE, Scopus, Web of Science Core Collection and Embase were searched without date restriction up to May 2026; seminal earlier publications identified through reference screening were retained where they remain the primary source for a given mechanism. Search terms combined descriptors for exposure and for mechanism, of the type (“berberine” OR “goldenseal” OR “Coptis” OR “Berberis”) AND (“drug interaction” OR “pharmacokinetic” OR “CYP3A4” OR “CYP2D6” OR “P-glycoprotein” OR “OCT2” OR “MATE1” OR “QT” OR “hERG”). Targeted searches were added for individual victim drugs, such as cyclosporine, metformin, warfarin and midazolam [65,71,117]. Reference lists of relevant articles were screened manually to identify additional sources.

We included in vitro studies on enzyme and transport systems, preclinical models, clinical pharmacokinetic/pharmacodynamic studies, case reports and case series, systematic reviews, and regulatory documents relevant to berberine’s interaction potential [126,127]. We excluded works without data on interactions or mechanisms, conference abstracts without verifiable data (except relevant safety signals, marked as such) and the purely commercial literature [125]. Data were extracted descriptively (mechanism, experimental system, victim drug, effect size, and clinical relevance) and synthesized thematically across the four mechanistic axes and the exposure modifier. The quality and transferability of each piece of evidence were appraised qualitatively, bearing in mind that, in preclinical models, berberine concentrations may exceed human systemic exposure [48,68]. Figure 1 and Figure 2 were prepared by the authors using Python (version 3.11) with the Matplotlib library (version 3.8.0, Matplotlib Development Team, open-source).

## 4. Conclusions

Berberine is not a harmless “natural” product but an active pharmacological perpetrator, with an interaction profile predictable from its pharmacology [35,37,111]. Low systemic exposure coexists with high local concentrations that inhibit CYP3A4/CYP2D6/CYP2C9, and can even induce CYP3A4 transcriptionally, and that modulate P-glycoprotein and organic cation transporters [35,59,65]; superimposed on these are additive pharmacodynamic effects, including hERG-channel block with QT prolongation [39,84]. The clinical anchor evidence on cyclosporine and the torsades de pointes signals compel a reassessment of the perception of harmlessness [39,118].

For the clinical pharmacist and for pharmacovigilance systems, the operational message is twofold [1,93]. Berberine must be actively sought in medication reconciliation and framed within a risk-stratification framework oriented toward drugs with a narrow therapeutic index and toward the multiple metabolic self-medication of the GLP-1 era [2,47]. The taxonomy of mechanisms, across four axes, together with the exposure modifier and the risk-stratification framework proposed here, provides a reproducible reasoning tool, intended to turn a fragmented field into a coherent safety practice [41,67]. The regulatory frame is shifting at the same time: national rules across Europe differ by more than an order of magnitude, and the European assessment under way has so far been unable to establish a safe intake level, so the dose a patient takes is currently determined as much by where the product was bought as by what the label states [114,115,116].

## Figures and Tables

**Table 3:** Risk-stratification framework for berberine interactions (perpetrator potency × victim vulnerability × patient vulnerability). INR = international normalized ratio; ECG = electrocardiogram.

Risk Level	Typical Situation	Recommended Course of Action
High (avoid the combination)	Victim with a narrow therapeutic index and a clear dominant mechanism (cyclosporine/tacrolimus, warfarin, digoxin, QT-prolonging drugs) in a vulnerable patient [40,87,118]	Discourage berberine use; if indispensable, only under supervision, with monitoring of concentrations/INR/ECG and dose adjustment of the victim drug [94,118]
Moderate (active monitoring)	Antidiabetics, antihypertensives, statins, CYP2D6/CYP3A4 substrates without a critical therapeutic index [35,36,59]	Educate the patient about signs of hypoglycemia/hypotension/myopathy; temporal separation of administration; targeted monitoring [2,30]
Low (standard caution)	Drugs without a relevant metabolic/transporter pathway and without pharmacodynamic additivity [1,2]	Record use in medication reconciliation; reassess at symptom onset or when the regimen changes [1,2]

## Data Availability

No new data were created or analyzed in this study. Data sharing is not applicable to this article.

## Abbreviations

The following abbreviations are used in this manuscript:

AMPK	AMP-activated protein kinase
APD	action-potential duration
AUC	area under the concentration–time curve
Cmax	peak plasma concentration
Cmin	trough plasma concentration
CYP	cytochrome P450
ECG	electrocardiogram
GI	gastrointestinal
GLP-1	glucagon-like peptide-1
hERG	human Ether-à-go-go-Related Gene
IC50	half-maximal inhibitory concentration
IKr	rapid delayed rectifier potassium current
IKs	slow delayed rectifier potassium current
INR	international normalized ratio
MATE1	multidrug and toxin extrusion protein 1
MDR1	multidrug resistance protein 1
mTOR	mechanistic target of rapamycin
NR	not reported
OCT	organic cation transporter
PBPK	physiologically based pharmacokinetic
PCSK9	proprotein convertase subtilisin/kexin type 9
P-gp	P-glycoprotein
PXR	pregnane X receptor
QT	QT interval
SANRA	Scale for the Assessment of Narrative Review Articles
SCFA	short-chain fatty acid
TdP	torsades de pointes
TPGS	D-α-tocopheryl polyethylene glycol 1000 succinate
